# HuNT: A Reference Transcriptome Atlas for Human Normal Tissues

**DOI:** 10.1007/s43657-025-00250-9

**Published:** 2026-05-18

**Authors:** KaiLin Tang, XinYan Xu, XinYue Tang, XinWen Wu, ZiHao Li, Jian Gao, ShiYue Zhang, SaiFeng Mo, ZiKun Chen, Mou Zhang, Hao Ding, Yao Shi, YiHeng Wang, YiXin Chen, MoHan Chen, ZhiWei Cao

**Affiliations:** 1 School of Life Sciences and Technology, Tongji University, Shanghai, 200092 China; 2 School of Life Sciences, Fudan University, Shanghai, 200441 China; 3 International Human Phenome Institutes (Shanghai), Shanghai, 200441 China; 4 Department of Thoracic Surgery and State Key Laboratory of Genetic Engineering, Fudan University Shanghai Cancer Center, 200032 Shanghai, China; 5 Institute of Thoracic Oncology, Fudan University, 200032 Shanghai, China; 6 Department of Oncology, Shanghai Medical College, Fudan University, 200032 Shanghai, China

**Keywords:** Transcriptome atlas, Human normal tissues, Integrated transcriptome, Tissue-specific genes, Housekeeping genes

## Abstract

With the rapid development of transcriptomics technology, gene expression profiles of normal human tissues have been frequently utilized as valuable references to study aging and disease mechanisms. Although individual databases have been established, most human tissues remain under-sampled. Meanwhile, profiles from RNA-seq and microarray platforms must be analyzed separately due to inherent distribution bias, further weakening the statistical power of health samples. Here, we constructed a comprehensive transcriptome atlas for human normal tissues (HuNT) containing 29,769 samples covering 74 human normal tissue types or parts, 70% and 37% larger than those in the well-known GTEx database in terms of sample size and tissue diversity, respectively. More importantly, instead of separate calculation, integrative analysis has been enabled for hybrid samples across RNA-seq and microarray profiles per tissue type. Based on the largest human sample collection so far, HuNT provides the latest information on: (1) tissue-specific genes, (2) stably and highly expressed housekeeping genes for each tissue, (3) gene expression landscape across all human tissues, and (4) top correlated genes for a query in a specified tissue. A case study of four widely-acknowledged biomarkers is also illustrated in the paper. With future data accumulation, HuNT may be useful to provide not only gene expression landscapes in human normal tissues, but also benchmarks to derive interesting marker genes and even therapeutic targets. HuNT is now freely accessible at http://hunt.badd-cao.net/.

**Supplementary Information:**

The online version contains supplementary material available at https://doi.org/10.1007/s43657-025-00250-9.

## Introduction

The past decades have witnessed the rapid development and application of transcriptome technology. A large amount of transcriptomics data has been produced and stored in public resources such as Gene Expression Omnibus (GEO) (Clough et al. 2023), Expression Atlas (George et al. 2023), Gene Expression Nebulas (GEN) (Zhang et al. 2022), etc. Among these, those of human normal tissues are extremely important and can be used as valuable references to study disease and aging mechanisms (Tang et al. 2017).

Earlier in 2013, the GTEx project initiated a pioneering work to sequence multiple "non-diseased" tissues obtained from recently deceased donors (Lonsdale et al. 2013). As the largest database to date, GTEx has collected RNA sequencing (RNA-seq) data from 17,452 samples across 54 human tissues according to its V8 release. Then, the TISSUES database was reported, focusing on human and three other species, with a confidence score developed to assess cross-species and cross-technology correlations (Santos et al. 2015). The updated TISSUES2.0 included 21 major human normal tissues covering 64,469 protein-tissue pairs based on microarray and RNA-seq (Palasca et al. 2018). Aiming to compare gene expression patterns between species, the Bgee database consolidated expression data from 29 species derived from various technologies, covering 58 human normal tissues and 11,075 human samples (Bastian et al. 2021). In the previous year, the Tedd database, focusing on developmental periods of humans and model organisms, collected RNA-seq data from 2760 bulk samples for human developmental tissues (Zhou et al. 2023). Among the above, the GTEx project alone has been cited more than 4600 times in studies on biomarkers (Demanelis et al. 2020), disease subtyping (Ling et al. 2020), etc., highlighting the significance of health control references.

It is recognized that the more samples are pooled together for the same tissue, the more reliable results can be expected statistically. Yet, a database review found that most human tissues remain under-sampled. Taking the largest database, GTEx, for instance, the sample size per tissue was noted to be highly heterogeneous, with the largest number of samples being 803 for muscle and only five samples for the kidney medulla. Secondly, technological biases arising from different sequencing platforms, typically RNA-seq and microarray technologies, posed a significant challenge for integrative analysis across RNA-seq and microarray data. In this study, we present HuNT, a reference transcriptome atlas for human normal tissues. By curating data from various sources such as GEO, ArrayExpress, TCGA, GTEx, and literature, we compiled a comprehensive atlas. Importantly, a state of the art (SOTA) method, Rank-in, was applied to reduce the data variability across RNA-seq and microarray platforms (Tang et al. 2021), allowing all samples for the same tissue to be mixed and analyzed to increase sample size and statistical power. The current version of HuNT comprises 29,769 normal samples covering 74 human tissue types/parts. Additionally, the most stably expressed housekeeping genes were identified for each tissue, together with tissue-specific genes, and correlated genes for each tissue. This database is now freely accessible at http://hunt.badd-cao.net/http://HuNT.badd-cao.net/.

## Methods

### Curation of Expression Data

Microarray and RNA-seq data of human normal tissues were derived from GEO, ArrayExpress, GTEx, TCGA (Cline et al. 2013), and the literature indexed in PubMed, with keywords such as "tissue" , "Homo sapiens", and "control/normal/match/health/adjacent", etc. Considering the coverage of microarray platforms and the number of genes shared with RNA-seq, only Affymetrix platforms were selected when collecting microarray data.

Expression datasets with over five samples per tissue and more than 10,000 genes were included. In order to avoid redundancies between the GEO and ArrayExpress databases, repetitive samples whose sample ID format was E-GEOD-n were removed.

For RNA-seq, the expression matrix of Fragments Per Kilobase of transcripts per Million mapped reads (FPKM) or Transcripts Per Million (TPM) was obtained, while for microarray, an expression matrix of intensity values was obtained. Donor information such as age and gender was also curated if available.

### Tissue Classification

In HuNT, the classification of tissues utilizes the established categorization frameworks of databases such as GTEx and Bgee. This classification of tissues is aligned with biological classifications and organ systems, adhering to internationally recognized nomenclature and classification standards. For tissues derived from public databases that do not specify a subgroup under a particular organ, they are labeled as "not annotated" under this organ within HuNT.

### Integration of the Hybrid Microarray and RNA-Seq Profiles

Zero-expressed genes were first removed across all samples of tissues. Then, the microarray data were log2-transdormed and then normalized using Robust Multi-array Average (RMA) (Teo et al. 2016), and RNA-seq data were transformed into $\:{{log}}_{2}(FPKM+1)$ or ${{log}}_{2}(TPM+1)$.

For each tissue, genes are initially arranged based on the ascending order of their expression levels within each transcriptomic profile. Following this, the genes are divided into 100 distinct groups according to their rankings, with the same number of genes in each group.

Subsequently, the groups are assigned weights corresponding to the gradient of expression levels across gene groups. This process results in a weighted ranking system for the genes within each profile.

Next, Singular value decomposition is applied to the aggregated profiles to minimize variability arising from different experimental conditions or technical platforms. This step standardizes the data distribution, ensuring a consistent trend across both microarray and RNA-seq data. The hybrid profiles were integrated by the Rank-in method for each tissue.

Then, the gene expression vectors were normalized to a range of 0 to 100 for a sample as follows:

$$\:{S}_{X-normalized}=\frac{{S}_{X}-{S}_{min}}{{S}_{max}-{S}_{min}}*100$$

Where S_X_ indicates the Rank-in value of gene X in a sample after integration, and S_min_ and S_max_ indicate the minimum and the maximum Rank-in value in all samples.

### Tissue-specific Genes

Tissue-specific genes are selected by the Specificity Measure (SPM), which is defined in the literature (Pan et al. 2012). The SPM values are then normalized to a range of 0 to 1 using the maximum and minimum SPM values:

$$\:{SPM}_{X-normalized}=\frac{{SPM}_{X}-{SPM}_{min}}{{SPM}_{max}-{SPM}_{min}}$$

Where SPM_X_ indicates the SPM value of gene X in a tissue, and SPM_min_ and SPM_max_ indicate the minimum and the maximum SPM value of all genes in this tissue.

A higher SPM value indicates higher tissue specificity.

### Housekeeping Genes

Housekeeping genes are defined as those that are expressed at a generally high level and showing small variance across all samples in a tissue.

Detailed criteria are as follows:

1. The median Rank-in score in the tissue > 60.
2. The difference in Rank-in score between Q3 (upper quartile) and Q1 (lower quartile) < 1.

## Results

### Overview of HuNT

**Fig. 1:**
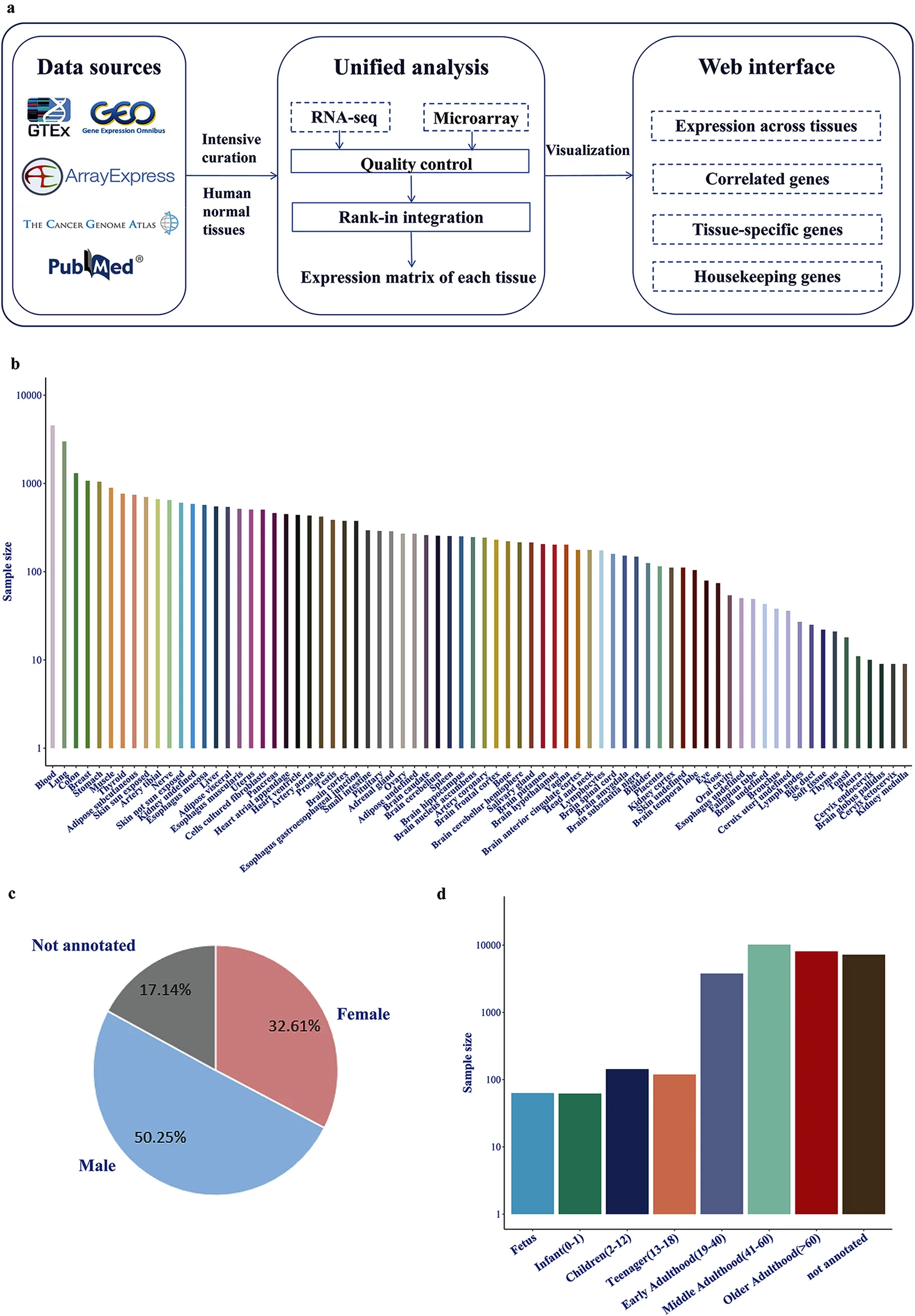
Overview of HuNT. **a** Pipeline overview; **b** Number of samples per tissue; **c** Gender proportion; **d** The sample size at different ages

As Fig. 1a shows, HuNT transcriptomic datasets were curated from different sources. After data cleaning, all samples of RNA-seq and microarray data from a tissue were consolidated by a state-of-the-art algorithm, Rank-in, to derive a unified expression matrix. Then, various statistics can be calculated based on the largest sample set so far, to identify marker genes or housekeeping genes for each tissue as well as across different tissues.

A total of 9960 datasets were initially retrieved, and 408 were finally included in HuNT. The current HuNT contains 29,769 human samples from 74 normal tissues with a total of 22,932 genes. The sample abundance per tissue is listed in Fig. 1b. Over 1000 samples were collected for each tissue of blood, lung, colon, stomach, and breast, peaking at 4547 samples in blood. Among the 74 tissues, 56 (approximately 76%) contain more than 100 samples. According to gender and age distribution (Fig. 1c and d), half of the samples were taken from male donors, and the donor age ranges from fetuses to over 60 years old.

### Peer Comparison

**Fig. 2:**
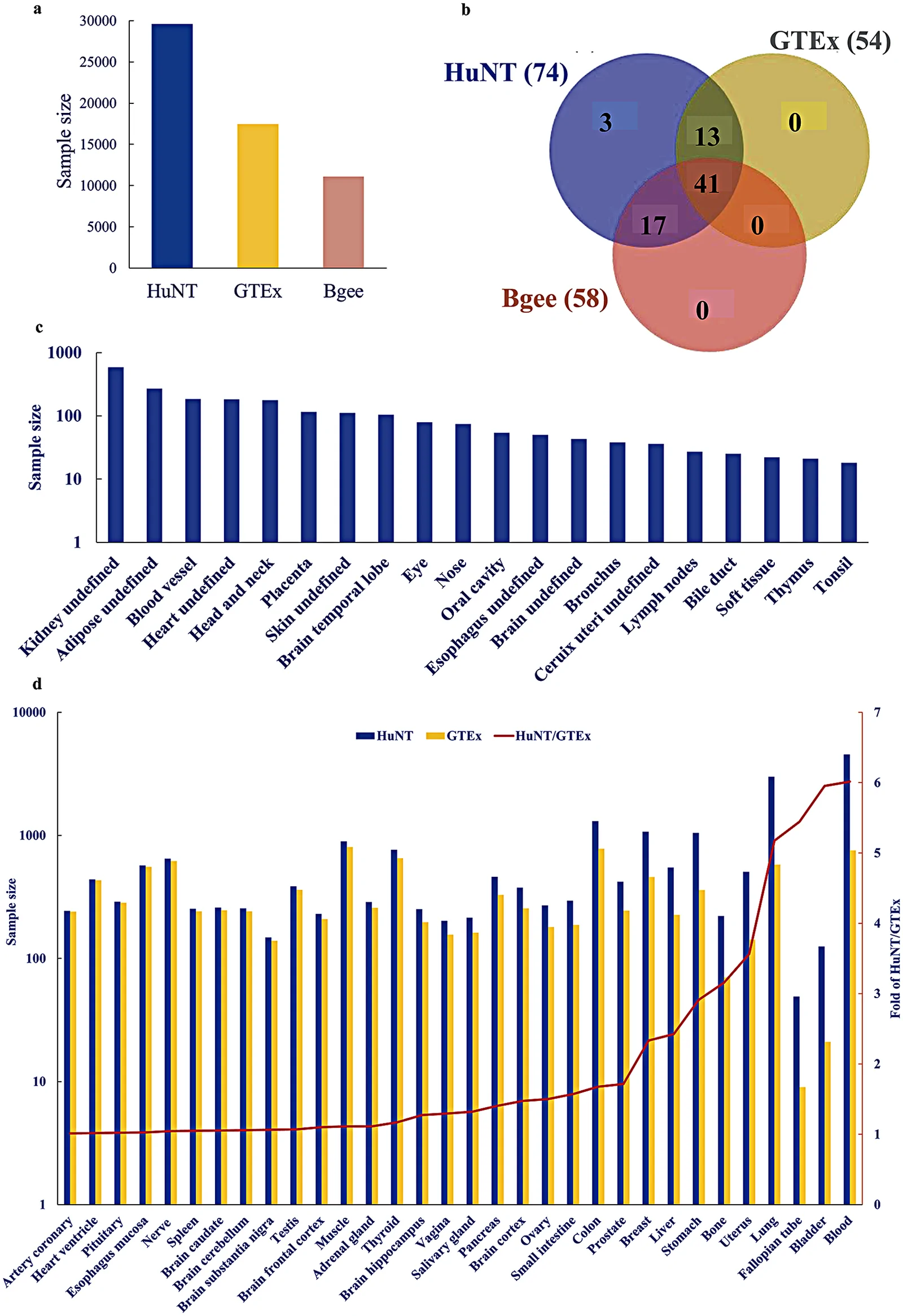
Comparison with peers. **a** Sample size of HuNT, GTEx, and Bgee; **b** Venn diagrams of tissues in HuNT, GTEx and Bgee; **c** Newly appended tissues in HuNT compared to GTEx; **d** Sample size and expansion fold of HuNT/GTEx for 36 tissues

HuNT details were compared with those of peer databases (Fig. 2a and b). Before HuNT, GTEx has been regarded as the largest and most well-known transcriptomic database for human normal tissues, although it only collects RNA-seq data. Compared to GTEx, HuNT expanded sample size and tissue diversity by 70% and 37%, respectively. Additionally, HuNT collected 20 new tissues that were missing in GTEx, such as kidney parts, adipose, head and neck, placenta, etc. (Fig. 2c). On the 54 tissues of GTEx, HuNT collected more samples than GTEx for 36 tissues. Sample expansion fold (HuNT/GTEx) was calculated for each of the 36 tissues, as shown in Fig. 2d. Taking blood as an example, the sample size increased from 755 in GTEx to 4547 in HuNT, with an expanding fold of 6.0. Healthy bladder was also detected with a large expansion fold of 5.95 (from 21 to 125), which is generally hard to obtain in clinical practice.

### Web Interface of HuNT

**Fig. 3:**
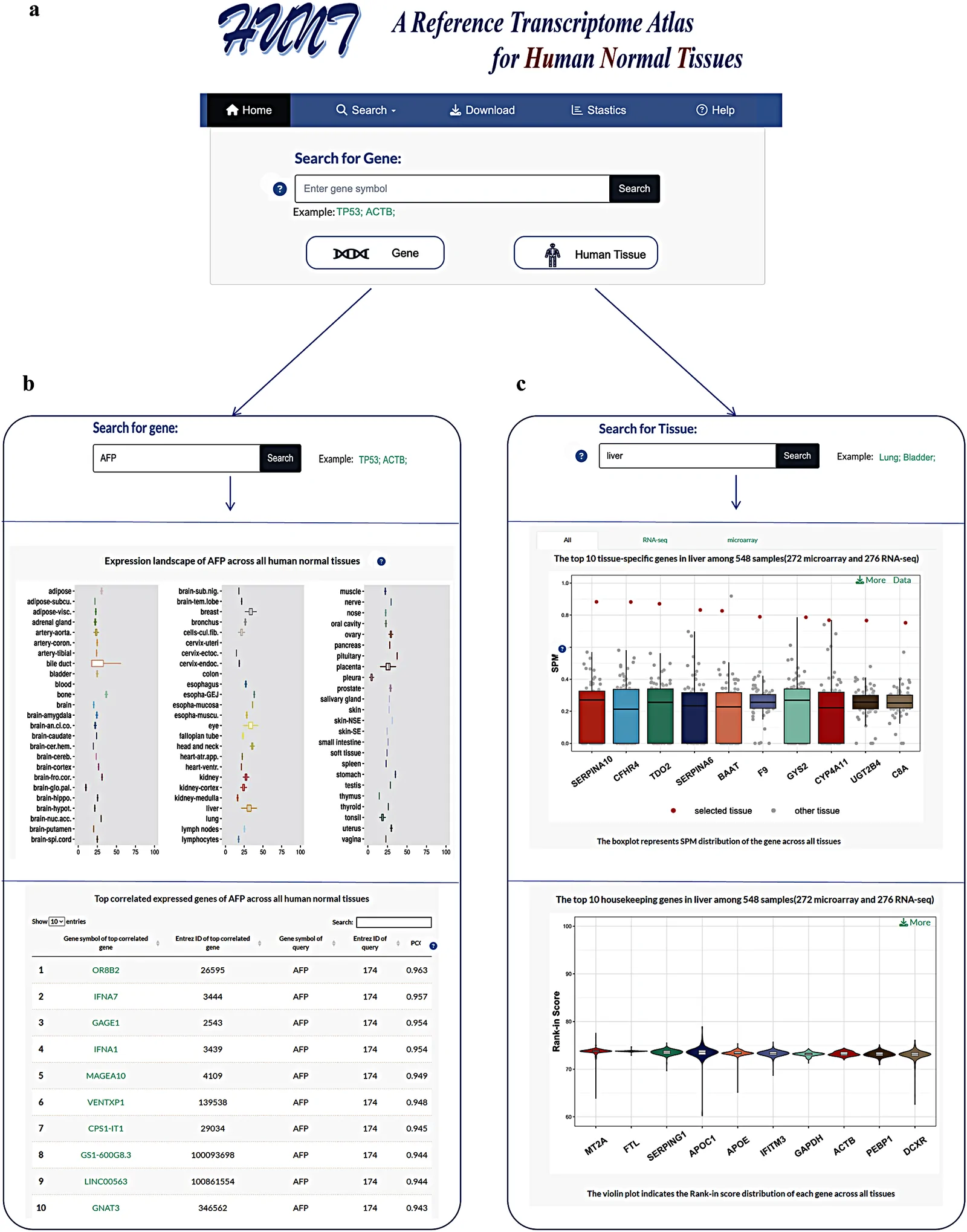
Web interface of HuNT. **a** HuNT homepage; **b** Screenshots of gene search taking *AFP* as an example; **c** Screenshots of tissue search taking liver as an example

A user-friendly interface was designed to search the gene expression landscape across different human tissue samples (Fig. 3a). Users can input a gene symbol to explore the expression variation among all samples per tissue. Top 20 correlated genes with the query gene were also listed according to Pearson correlation coefficient (PCC) across 74 tissues. The boxplot in Fig. 3b shows the results of the *Alpha-Fetoprotein (AFP)* query. Moreover, the top correlated genes were also shown with similar expression patterns to *AFP* across different tissues. Alternatively, users can query a tissue to obtain tissue-specific genes and stably-expressed genes in each tissue (Fig. 3c). Users can also download the full list of these genes for further research.

All data in HuNT are accessible via the "Download" button. A hyperlink to the original data source is also available on the website.

### Example Illustration

**Fig. 4:**
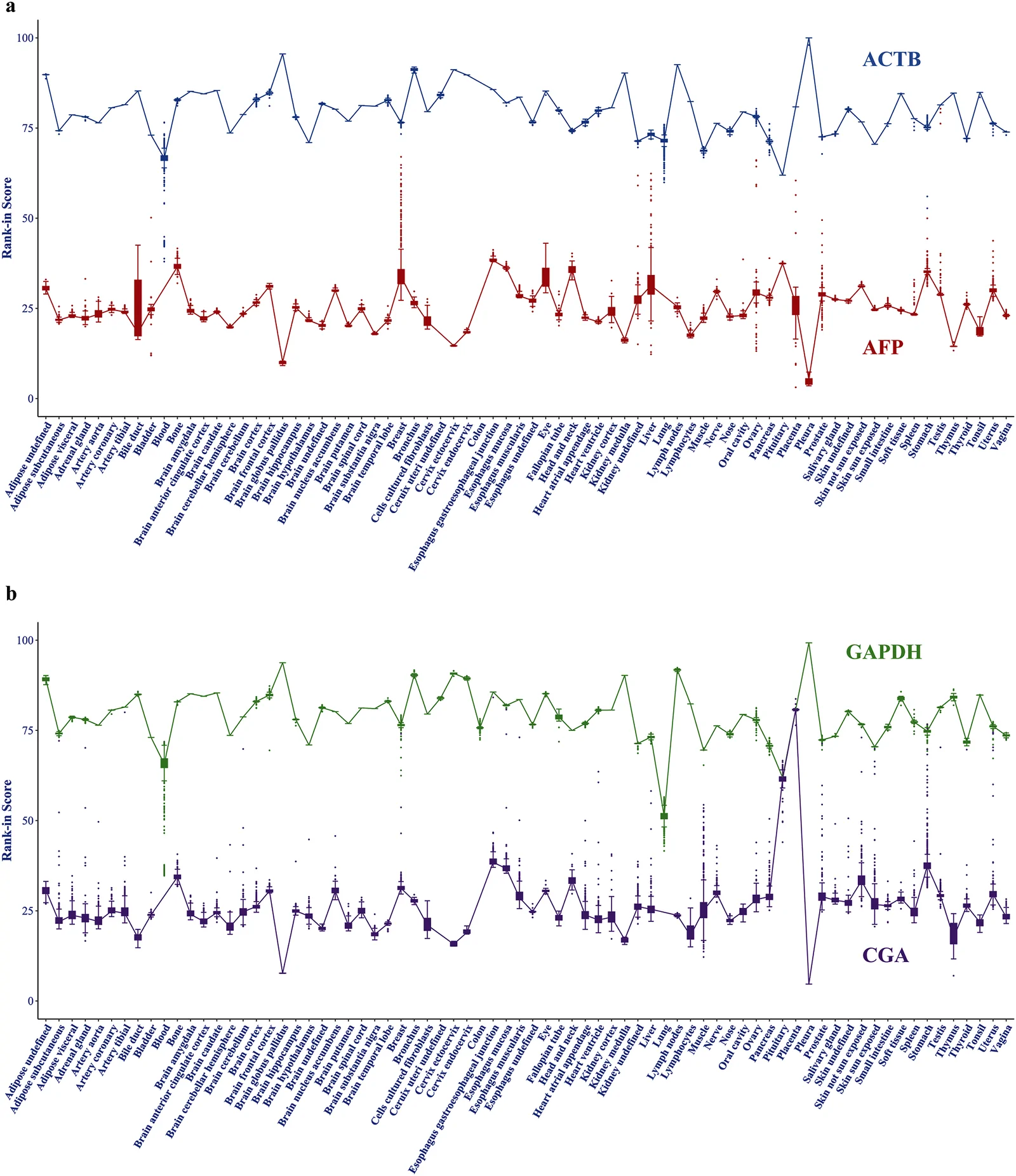
Gene expression landscape of disease marker genes and housekeeping genes. **a** The Rank-in score boxplots of *ACTB* and *AFP* for each tissue type/part; **b** The Rank-in score boxplots of *GAPDH* and *CGA* for each tissue type/part

To illustrate the usage of HuNT, four example genes were selected, including two disease marker genes (*AFP*,* CGA*), and two commonly used positive controls for gene expression (*GAPDH*,* ACTB*) (Zhang, Zhou, et al. 2024). *AFP*, a glycoprotein originally found in human fetuses, has been well-known as a marker for the diagnosis and prognosis of hepatocellular carcinoma (HCC) (Johnson et al. 2022), with a high expression level in tumor samples (Zhao et al. 2022). In addition, *AFP* is often used as an informative marker for gastric cancer, colonic adenocarcinoma, renal cell carcinoma, and so on (Ma et al. 2023). *CGA* (also known as *HCG*), a glycoprotein normally produced mainly by the placenta during pregnancy and the pituitary gland, is a noted marker gene for germ cell tumors (GCTs) (Pedrazzoli et al. 2021), with elevated levels frequently found in GCTs, while a moderate increase was observed in cancers of cervix, uterus and vulva, lung cancer, prostate cancer, and gastrointestinal tract cancers (Desai and Guddati 2023). Generally, *AFP* and *CGA* are expected to be expressed at very low levels in normal healthy tissues, while *GAPDH* and *ACTB* are expressed at high levels. The expression boxplots of the four genes were displayed in Fig. 4, together with their distribution.

As expected, housekeeping genes *GAPDH* and *ACTB* are expressed at relatively high and stable levels with low variance across all tissues. However, marked variability still exists among different tissues even for the same gene. For instance, *GAPDH* is expressed at the highest level in pleural tissue in 11 samples (median 99.27, standard deviation 0.00), and at the lowest level in lung tissue in 2999 samples (median 51, standard deviation 1.37). In contrast, disease markers *AFP* and *CGA* showed low expression across different tissue types/parts, but large inter-individual variance within the same tissue. Interestingly, a sharp peak was noted for *CGA* in placenta (median of 80.75 and standard deviation of 0.56), which is reasonable.

Previously, microarray and RNA-seq data of the same tissues could only be analyzed separately, and discrepancies and even conflicts were often noted between the results. Again, taking *GAPDH* in the lung as an example, there were 829 RNA-seq and 2162 microarray profiles from lung samples. When only the source microarray data were analyzed for HuNT, *GAPDH* showed the lowest expression with large inter-individual variance in lung compared to the other tissues. When switching to RNA-seq source data, this conclusion no longer holds (Fig. S1). The discrepancy may be caused by inter-individual genetic variation and/or non-biological effects across technological platforms. Here, HuNT integrated all the samples by minimizing technological bias, and provides a one-stop platform for gene expression landscape based on a large number of samples of normal tissues.

## Discussion

Human normal tissues play fundamental roles in studying disease mechanisms as reference controls. More normal tissue samples help to derive more statistically significant results. Here, HuNT was set up as the largest transcriptomic atlas so far, comprising 29,769 samples across 74 human normal tissues by manually collating data from available databases and the literature. Different from other databases, HuNT collected both tissues from healthy donors and pathologically normal tissues from patients, and included both RNA-seq and microarray profiles to increase sample size and tissue diversity. Tissue samples were mostly derived from healthy individuals, with less than 10% from peritumoral tissues of clinical patients.

Importantly, HuNT enables consolidated analysis of freely blended data from microarray and RNA-seq samples driven by a state-of-the-art algorithm, Rank-in, proposed in 2021. Rank-in enables integration through a weighted internal ranking of gene expression, coupled with singular value decomposition (SVD), thereby mitigating the inherent variability in data distribution between microarray and RNA-seq technologies. Having shown potential in the identification of co-diagnostic genes from different public datasets (Zhang et al. 2023), it has been used to "enhance the accuracy and effectiveness of biomarker discovery through the integration of data from RNA-Seq and microarray experiments" (Janyasupab et al. 2023).

Of note, Rank-in integration only uses those overlapping genes shared between microarray and RNA-seq samples for each tissue. Microarray technology relies on predetermined gene probes, whereas RNA-seq provides a broader perspective on the transcriptome without the need for pre-defined probes. The number of genes detected by transcriptomics technology may vary with the sequencing depth and designed primers of different platforms. Accordingly, the more samples are integrated, the fewer genes are likely to overlap. That is why we purposely balanced the sample size and gene number. Though zero-expressed genes were filtered out at the initial stage, normalization of Rank-in values produces zero values again, indicating the lowest expression level. Meanwhile, a large gap between microarray and RNA-seq sample size may influence the integration, and the conclusion tends to skew towards the larger side.

In addition, integration of more samples was frequently accompanied by larger inter-individual variance, which may affect the results of tissue-specific genes and housekeeping genes. In this study, a housekeeping gene was calculated across all samples of the same tissue as being highly expressed with low expression variation. In some other studies, however, it was defined across different tissues. The housekeeping genes derived differently may not necessarily overlap as much as expected. In fact, the perplexing behaviors of these genes have long been debated. As early as in 2015, the designation of housekeeping genes was reported to be inappropriate across all tissue types (Zhang et al. 2015), as many so-called housekeeping ones exhibited large expression variability in specific tissues (Joshi et al. 2022). Regarding tissue-specific genes, we found that they were often related to tissue diversity. The more diverse the tissues are, the more tissue-specific genes can be identified for each tissue. Further, the tissue-specific genes were validated in the GTEx cohort, with two examples of *SFTPC* (specific in lung) and *SMTNL1* (specific in muscle) as illustrated in Fig. S2. It can be observed that the two genes identified by HuNT are uniquely and highly expressed in the corresponding tissue types within the GTEx dataset, thereby confirming their tissue-specific roles. Similarly, the tissue-specific genes derived from GTEx calculations have also been validated in HuNT, even in tissues where the majority of samples were not from GTEx. For example, *CLEC4D* and *TAGAP* (specific in 755 blood samples) are highly expressed in GTEx. Though it is not the top tissue-specific gene of blood in HuNT, the expression of *CLEC4D* and *TAGAP* in blood (4541 samples) is also the highest among 74 tissues in HuNT (Fig. S3).

## Conclusion

HuNT, a reference transcriptome atlas for human normal tissues, was established by curating both RNA-seq and microarray profiles of 29,769 samples for 74 human normal tissues, emerging as the largest database of human normal tissues. HuNT offers valuable information regarding: (i) the baseline gene expression landscape across 74 normal tissues/tissue parts, (ii) the correlated gene expression patterns across 29,769 human healthy samples, and (iii) the tissue-specific and housekeeping genes in each tissue/tissue part. HuNT may be useful not only in providing gene expression patterns in normal tissues, but also in serving as a benchmark for pathological tissues in the discovery of interesting biomarkers or therapeutic targets. Future improvements will focus on the accumulation of more tissue samples, as well as detailed classification of tissue parts.

## Electronic Supplementary Material

Below is the link to the electronic supplementary material.

Supplementary Material 1

Supplementary Material 2

Supplementary Material 3

Supplementary Material 4

## Data Availability

All data curated and integrated during this study are available in HuNT (http://hunt.badd-cao.net/).

## Abbreviations

GTEx: Genotype-Tissue Expression
RNA-seq: RNA Sequencing
Bgee: A database for retrieval and comparison of gene expression patterns across multiple animal species
GEO: Gene Expression Omnibus
TCGA: The Cancer Genome Atlas
SOTA: State of the art
FPKM: Fragments Per Kilobase of transcript per Million mapped reads
TPM: Transcripts Per Million
RMA: Robust Multi-array Average
SPM: Specificity Measure
PCC: Pearson correlation coefficient
AFP: Alpha-Fetoprotein
CGA: Glycoprotein hormones, alpha polypeptide
GAPDH: Glyceraldehyde-3-phosphate dehydrogenase
ACTB: Actin Beta
HCG: Human Chorionic Gonadotropin
SFTPC: Surfactant protein C
SMTNL1: Smoothelin like 1, calponin homology-associated smooth muscle protein CLEC4D: C-type lectin domain family 4 member D
TAGAP T: Cell activation RhoGTPase activating protein

## References

[CR1] Bastian FB, Roux J, Niknejad A et al (2021) The Bgee suite: integrated curated expression atlas and comparative transcriptomics in animals. Nucleic Acids Res 49(D1):D831–D847. 10.1093/nar/gkaa793 33037820 PMC7778977

[CR2] Cline MS, Craft B, Swatloski T et al (2013) Exploring TCGA Pan-Cancer data at the UCSC cancer genomics browser. Scientific Reports 3:2652 10.1038/srep02652 PMC3788369 24084870

[CR3] Clough E, Barrett T, Wilhite SE et al (2023) NCBI GEO: archive for gene expression and epigenomics data sets: 23-year update. Nucleic Acids Res. 10.1093/nar/gkad965 PMC10767856 37933855

[CR4] Demanelis K, Jasmine F, Chen LS et al (2020) Determinants of telomere length across human tissues. Science 369(6509):1333–. 10.1126/science.aaz6876 PMC8108546 32913074

[CR5] Desai S, Guddati AK (2023) Carcinoembryonic antigen, carbohydrate antigen 19 – 9, cancer antigen 125, Prostate-Specific antigen and other cancer markers: A primer on commonly used cancer markers. World J Oncol 14(1):4–14. 10.14740/wjon1425 36895994 PMC9990734

[CR6] George N, Fexova S, Fuentes AM et al (2023) Expression atlas update: insights from sequencing data at both bulk and single cell level. Nucleic Acids Res. 10.1093/nar/gkad1021 PMC10767917 37992296

[CR7] Janyasupab P, Suratanee A, Plaimas K (2023) GeneCompete: an integrative tool of a novel union algorithm with various ranking techniques for multiple gene expression data. Peerj Comput Sci 9:e1686. 10.7717/peerj-cs.1686 PMC10703088 38077583

[CR8] Johnson P, Zhou Q, Dao DY et al (2022) Circulating biomarkers in the diagnosis and management of hepatocellular carcinoma. Nat Reviews Gastroenterol Hepatol 19(10):670–681. 10.1038/s41575-022-00620-y 35676420

[CR9] Joshi CJ, Ke W, Drangowska-Way A et al (2022) What are housekeeping genes? PLoS Comput Biol 18(7):e1010295. 10.1371/journal.pcbi.1010295 35830477 PMC9312424

[CR10] Ling JP, Wilks C, Charles R et al (2020) ASCOT identifies key regulators of neuronal subtype-specific splicing. Nat Commun 11(1). 10.1038/s41467-019-14020-5 PMC6952364 31919425

[CR11] Lonsdale J, Thomas J, Salvatore M et al (2013) The Genotype-Tissue expression (GTEx) project. Nat Genet 45(6):580–585. 10.1038/ng.2653 23715323 PMC4010069

[CR12] Ma CY, Jin YXZ, Wang YH et al (2023) Beyond liver cancer, more application scenarios for alpha-fetoprotein in clinical practice. Front Oncol 13. 10.3389/fonc.2023.1231420 PMC10540843 37781207

[CR13] Palasca O, Santos A, Stolte C et al (2018) TISSUES 2.0: an integrative web resource on mammalian tissue expression. Database-the Journal of Biological Databases and Curation. 10.1093/database/bay028 PMC5808782 29617745

[CR14] Pan JB, Hu SC, Wang H et al (2012) PaGeFinder: quantitative identification of Spatiotemporal pattern genes. Bioinformatics 28(11):1544–1545. 10.1093/bioinformatics/bts169 22492640 PMC3356841

[CR15] Pedrazzoli P, Rosti G, Soresini E et al (2021) Serum tumour markers in germ cell tumours: from diagnosis to cure. Crit Rev Oncol Hematol 159:103224 10.1016/j.critrevonc.2021.103224 33493632

[CR16] Santos A, Tsafou K, Stolte C et al (2015) Comprehensive comparison of large-scale tissue expression datasets. Peerj 3:e1054 . 10.7717/peerj.1054 PMC4493645 26157623

[CR18] Tang ZF, Li CW, Kang BX et al (2017) GEPIA: a web server for cancer and normal gene expression profiling and interactive analyses. Nucleic Acids Res 45(W1):W98–W102. 10.1093/nar/gkx247 28407145 PMC5570223

[CR17] Tang KL, Ji XJ, Zhou MD et al (2021) Rank-in: enabling integrative analysis across microarray and RNA-seq for cancer. Nucleic Acids Res 49(17). 10.1093/nar/gkab554 PMC8464058 34214174

[CR19] Teo ZL, Savas P, Loi S (2016) Gene Expression Analysis: Current Methods. In: Lakhani SR, Fox SB (eds) Molecular Pathology in Cancer Research, Springer New York, New York, NY, 107–136. 10.1007/978-1-4939-6643-1_6

[CR20] Zhang Y, Li D, Sun B (2015) Do housekeeping genes exist? PLoS ONE 10(5):e0123691. 10.1371/journal.pone.0123691 25970694 PMC4430495

[CR21] Zhang Y, Li JW, Chen LH et al (2023) Identification of co-diagnostic effect genes for aortic dissection and metabolic syndrome by multiple machine learning algorithms. Sci Rep 13(1). 10.1038/s41598-023-41017-4 PMC10491590 37684281

[CR25] Zhang Y, Zhou Y, Zhou Y et al (2024) TheMarker: a comprehensive database of therapeutic biomarkers. Nucleic Acids Res 52(D1):D1450-D1464. 10.1093/nar/gkad862 PMC10767989 37850638

[CR22] Zhang YS, Zou D, Zhu TT et al (2022) Gene expression nebulas (GEN): a comprehensive data portal integrating transcriptomic profiles across multiple species at both bulk and single-cell levels. Nucleic Acids Res 50(D1):D1016–D1024. 10.1093/nar/gkab878 34591957 PMC8728231

[CR23] Zhao KL, Zhou XQ, Xiao YC et al (2022) Research progress in Alpha-fetoprotein-induced immunosuppression of liver cancer. Mini-Reviews Med Chem 22(17):2237–2243. 10.2174/1389557522666220218124816 35184712

[CR24] Zhou ZH, Tan C, Chau MHK et al (2023) TEDD: a database of Temporal gene expression patterns during multiple developmental periods in human and model organisms. Nucleic Acids Res 51(D1):D1168–D1178. 10.1093/nar/gkac978 36350663 PMC9825605

